# Early Initiation of Vestibular Therapy Following Sports-Related Concussions: A Retrospective Cohort Study

**DOI:** 10.7759/cureus.39764

**Published:** 2023-05-31

**Authors:** Benjamin Ferry, Gary Means, Cynthia Green, Thomas Risoli, Corina Martinez, Rock P Vomer, Emily Reinke, Courtney Pyles, Jeffrey Bytomski

**Affiliations:** 1 Department of Family Medicine, Division of Sports Medicine, Liberty University College of Osteopathic Medicine, Lynchburg, USA; 2 Department of Family Medicine and Community Health/Department of Orthopaedics, Division of Sports Medicine, Duke University, Durham, USA; 3 Department of Biostatistics and Bioinformatics, Duke University, Durham, USA; 4 Department of Rehabilitation Services, Duke University, Durham, USA; 5 Family Medicine, Mayo Clinic Jacksonville Campus, Jacksonville, USA; 6 Department of Orthopaedics, Duke University, Durham, USA

**Keywords:** vestibular rehabilitation, adult concussion recovery, concussion recovery, sports related concussion, concussion

## Abstract

Background: Vestibular dysfunction is common following sports-related concussions (SRC). Within the current practice, it is theorized that patients with vestibular dysfunction as sequelae of sports-related concussion have a prolonged recovery time compared to those without vestibular dysfunction.

Study method: A retrospective, cohort investigation of 282 subjects with sports-related concussions with vestibular dysfunction was conducted at The Sports Medicine Concussion Clinic, Duke University. The primary endpoint was the return-to-play (RTP) date.

Results: For every one-day increase in time from injury to initial vestibular therapy, the geometric mean time from injury to RTP increases by 1.02 days (exp{β}=1.02 days; 95% CI: 1.01, 1.02 days; p<0.001).

Conclusion: Our data suggest an association between the timing of vestibular therapy in SRC and a direct relationship to earlier recovery and return to sport.

## Introduction

Sports-related concussion (SRC) is a common injury among athletes at all levels. The symptoms associated with an SRC are heterogenous but include headaches, poor concentration, vertigo, and vision difficulties. Vestibular dysfunction is a common sequela of SRC occurring in greater than 50% of SRC patients [1]. Furthermore, dizziness occurring immediately after an injury, a specific symptom of possible vestibular function, has been found to be a predictor of prolonged recovery after SRC [2]. Of particular concern for students with SRC is the time lost from academic work following an SRC. High school and collegiate athletes frequently miss significant class time following an SRC, and some of the most common symptoms that limit a patient’s ability to return to academic work are vestibular symptoms causing dizziness, vertigo, nausea, and headaches [3].

The wide variety of concussion symptoms is frequently measured with the Post-Concussion Symptom Score [3]. This tool allows quantification of the broad spectrum of concussion symptoms with each patient encounter. Additionally, vestibular dysfunction in concussion patients can be accurately identified through the use of the Vestibular Oculomotor Screening (VOMS) test [1]. This simple six-step screening test can identify various types of vestibular dysfunction typically associated with sports-related concussions. Other factors associated with prolonged recovery from concussion include prior concussion, prior psychiatric illness, learning disability, and female gender [4].

Until recently, treatment for SRC involved a period of strict physical and cognitive rest prior to the initiation of any activity or specific therapy [5]. Treatment models have begun to incorporate earlier exercise and cognitive activity as tolerated, and these models seem to facilitate earlier return to play (RTP) for athletes [4]. Typically, patients with vestibular dysfunction after an SRC are treated with relative physical and cognitive rest followed by a progression of athletic and academic activity. Targeted vestibular therapy has traditionally not been utilized unless symptoms of vestibular dysfunction persist beyond the expected recovery timeframe (greater than 10-14 days in adults and greater than four weeks in children) [4,6]. Symptoms of vestibular dysfunction are associated with significant morbidity, including longer time-to-clearance for return to sport [3,7]. Based on this, it is suspected that vestibular therapy following SRC in patients with persistent vestibular dysfunction allows for faster rates of recovery and return to sport [8]. The impact of the initiation of vestibular therapy early in the post-injury course following SRC is unknown. The primary purpose of this study was to ascertain whether a correlation exists between early initiation of vestibular therapy following an SRC and earlier return-to-sport after injury.

Additionally, recent literature has begun to show a correlation between a variety of pre-injury factors and risk for prolonged recovery, some of which were detailed above. However, literature is still emerging regarding projection of recovery course based on a variety of other factors, including injury characteristics and findings on a variety of post-injury assessment tools. A 2021 study by Putukian et al. demonstrated that post-injury ImPACT symptom score, reaction time, and visual motor composite assessments were associated with delayed time until return-to-play (RTP) [9]. A 2017 study created a nomogram for the prediction of recovery at the six-month mark after mild traumatic brain injury [10]. However, to our knowledge, a nomogram for specific prediction of return-to-play time after sport-related concussion has not been previously created. An additional purpose of this study was to create a nomogram that would allow for clinician estimation of time to clearance for RTP after SCR based on a variety of clinical factors.

## Materials and methods

Design and participants

**Figure 1:**
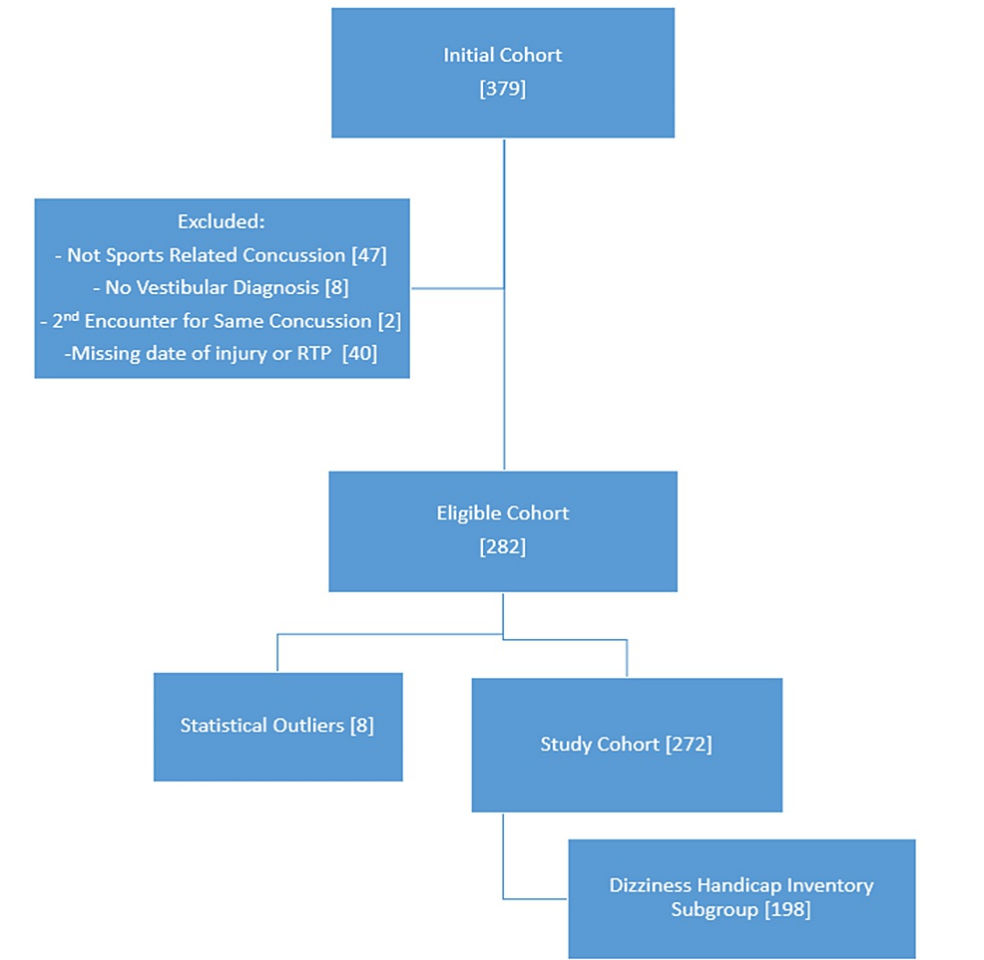
Chart review process The flow of chart review for inclusion and exclusion of subjects.

A retrospective, cohort investigation was conducted by chart review to evaluate patients who presented with SRC to the Sports Medicine Concussion Clinic at Duke University between January 2014 and December 2019. This study was approved by the Institutional Review Board (protocol number Pro00104023). The initial chart review was conducted for 379 patients who were evaluated in the Sports Medicine Concussion Clinic and referred to physical therapy (PT) (for vestibular therapy). Only those patients between the ages of 12-25 years (at the time of injury) with a medically diagnosed SRC were included in the analysis. Patients with non-SRCs (motor vehicle accidents, falls during an activity of daily living, etc) were excluded, as were patients without vestibular therapy referrals. Two patients were excluded as a duplicate record had been created for the same concussion. Patients without return-to-play dates and those lost to follow-up were also excluded (Figure 1). Additional exclusion criteria included the following: previously diagnosed neurological conditions or disorders, moderate or severe traumatic brain injury, preexisting vestibular disorder, and preexisting intracranial/cervical pathology; ultimately no additional patients were excluded based on these criteria. 

There were six patients who sustained two separate SRCs during the study period; these injuries/treatments occurred with sufficient temporal distance so that they were treated as independent observations and included in the analysis. Patient records matching the above criteria were identified using the Duke DEDUCE query tool [11]. This data was extracted from the electronic medical record and managed utilizing a RedCap database [12,13]. The RedCap database was maintained in a secure computing environment through PACE (Protected Analytics Computing Environment).

Measures

SRCs were identified through certified athletic trainers associated with schools, primary care providers, or urgent care/emergency department providers. These patients were then referred to the Sports Medicine Concussion Clinic and subsequently underwent a multimodal evaluation based initially upon Sports Concussion Assessment Tool 3 (SCAT3) and later SCAT5 [14,15]. If vestibular dysfunction was identified by the sports medicine physician during the multimodal evaluation, those patients were then referred for vestibular therapy treatment through the Sports Physical Therapy clinic. Physical therapy interventions were prescribed based on the symptoms provoked during the performance of the VOMS and SWAY [16,17]. Exercises included gaze stabilization, habituation, and balance activities that were progressed by modifying amplitude and velocity, the base of support (BOS), visual input, and external perturbations. Patients were instructed to perform exercises at sub-symptomatic velocity or amplitude to prevent provoking cervical or visual-based symptoms. Additional modifications were made to address any symptoms that were also related to cervical, visual, or autonomic impairments.

North Carolina mandates a specific return-to-play protocol following SRC. Initiation of this 5-stage protocol requires authorization by a licensed health care provider (LHCP) with concussion management experience. Once the protocol has been initiated, it can be monitored by authorized personnel; in the region of this study, that person was typically the associated school Licensed Athletic Trainer. Return-to-play date was determined through either explicit documentation of the actual return-to-play date as determined by correspondence with the parent or the school-based athletic trainer or through estimation of the return-to-play date based upon physician documentation of return-to-play protocol initiation. The number of days from the initial injury to the completion of the graduated return-to-play protocol was used for the statistical analyses.

Statistical analysis

Statistics were performed using SAS software version 9.4 (SAS Institute Inc., Cary, NC, USA) and R Studio (R version 3.6.2 "Dark and Stormy Night", 2019-12-12) with R package “ggplot2” [14]. Univariable and multivariable lognormal regression models were used to assess the relationship between time from injury to RTP and time from injury to initial vestibular therapy, controlling for covariates. We chose to model time from injury to return to sport as lognormal based on preliminary diagnostics of the distribution. We also decided to use the “sandwich” estimator for the variance to account for the heteroscedasticity. Time from injury to first physical therapy appointment was also modeled using restricted cubic spline modeling. For this model and cohort, restricted cubic spline modeling was completed with three knots placed at the 10th, 50th, and 90th percentiles. A nomogram was created using the multivariable logistic regression model from the time of injury to RTP.

A secondary sub-analysis, including only those patients with the Dizziness Handicap Index Score (DHI) at initial vestibular therapy appointment, was performed to measure its relationship with time from injury to return to sport. Sample sizes for individual sports were not sufficient to allow for inclusion in the multivariable analysis.

## Results

Distribution and frequencies for categorical variables are presented as counts and percentages for non-missing data, and continuous variables are presented as the median, the 25th and 75th percentiles, and the mean and standard deviations (Table 1). There were 53.3% female and 46.7% male patients in the sample. The rate of prior concussion was 31% (n=85), and the history of prior diagnosis of Attention Deficit Hyperactivity Disorder or other Diagnostic and Statistical Manual of Mental Disorders (DSM) diagnoses was 28.8% (n=79) (Table 1).

**Table 1:** Binary patient characteristics in the study population.

Characteristics	Total (N=274)
Sex	
Female	146 (53.3%)
Male	128 (46.7%)
History of a Prior Concussion	
No	189 (69.0%)
Yes	85 (31.0%)
History of a Prior Psychiatric Diagnosis	
No	195 (71.2%)
Yes	79 (28.8%)

**Figure 2:**
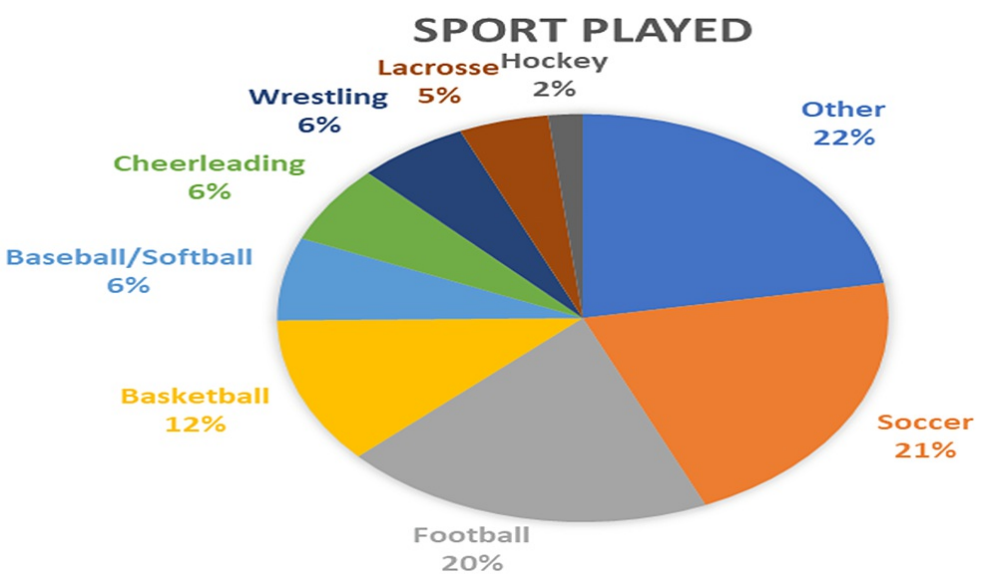
Type of sports breakdown. The pie chart represents the breakdown of sports played by the subjects.

A wide variety of sports were represented in the sample with the largest percentages belonging to soccer (21.2%, n=58), football (19.7%, n=54), and basketball (11.7%, n=32) (Figure 2).

Patient demographics ranged in age from 12 to 25 years (mean ± SD, 15.7 ± 2.2) at the time of injury. Out of the total, 91.6% of the patients were under the age of 18 years at the time of injury (Table 2).

**Table 2:** Continuous patient characteristics in the study population PT: Physical therapy

Characteristic (n=274)	Median	Q1,Q3	Mean (SD)
Age (Years)	15.0	14.0, 16.0	15.7 (2.2)
Time from Injury to Initial Evaluation (days)	5.0	3.0,9.0	12.0(26.7)
Number of Symptoms at Initial Evaluation	14.0	10.0,17.0	13.6(4.7)
Missing	19 (6.9%)		
Post-Concussion Symptom Scale Score at Initial Evaluation	35.5	22.0, 54.5	39.8 (22.4)
Missing	2 (0.7%)		
Time from Injury to Initial PT Visit (days)	8.0	6.0, 14.0	16.5 (27.7)
Number of Symptoms at Initial PT Visit	12.0	7.0, 17.0	11.6 (6.2)
Missing	1 (0.4%)		
Post-Concussion Symptom Scale Score at Initial PT Visit	23.0	11.0, 43.0	28.4 (22.6)
Missing	2 (0.7%)		
Dizziness Handicap Inventory Score at Initial PT Visit	29.5	12.0, 40.0	27.5 (18.9)
Missing	76 (27.7%)		
Time from Initial Evaluation to Initial PT (days)	3.0	1.0, 5.0	4.5 (5.7)
Time from Injury to Return to Sports (days)	26.5	17.0, 45.0	46.3 (56.5)

One hundred and seventy patients had clearly delineated RTP dates; the remaining 112 patients had RTP estimated based on physician release and North Carolina return-to-play protocol. The median time elapsed between the initial injury and the initial vestibular therapy appointment was 8.0 days (mean 16.5, SD 27.7). The median time from injury to RTP was 26.5 days.

Table 2 shows the results of the univariable and multivariable regression of time from injury to return to sport on patient characteristics. Beta estimates and 95% confidence intervals for univariable and multivariable results are shown in the table. Since the outcome was modeled on the log scale, the estimates were exponentiated to interpret the coefficients better to give the relative change in the geometric mean of our outcome. Of the various factors analyzed, female sex, time from injury to initial PT, and symptom score at the time of evaluation were found to be statistically significant in regard to prolonged time to RTP. Adjusting for the other characteristics, female athletes had an increase in the geometric mean of time from injury to RTS of a factor of 1.25 (exp{β}=1.25 days; 95% CI: 1.06, 1.46 days; p=0.006). Additionally, for every one-day increase in time from injury to initial PT, the geometric mean of time from injury to RTS increases by a factor of 1.02 days (exp{β}=1.02 days; 95% CI: 1.01, 1.02 days; p<0.001). The R-squared for this model was 0.40, which means that the model is explaining 40% of the total variance of time from injury to RTS. Lastly, the total symptom score at initial evaluation also was associated with an increase in the geometric mean of time from injury to RTS by a factor of 1.01 exp(β)=1.01 days; 95% CI: 1.01, 1.01 days; p<0.001).

Table 2 represents a univariable and multivariable regression of time from injury to RTS on time from injury to initial PT using restricted cubic splines. The association between time from injury to initial PT and time from injury to RTS is stable between the univariable and multivariable models. Adjusting for the other characteristics, from a 10 to 20-day increase in time from injury to initial PT, the geometric mean of time from injury to RTS increases by a factor of 1.60 days (exp{β}=1.60 days; 95% CI: 1.46, 1.76 days; p<0.001). The R-squared for this model was 0.49, meaning that the model explains 49% of the total variance of time from injury to RTS.

DHI sub-analysis

In this sub-analysis, patients were removed if they were missing their DHI score at the time of their PT. This resulted in the effective sample size being reduced from 274 to 198. In this model and cohort, adjusting for the other characteristics, for every one-day increase in time from injury to initial PT, the geometric mean of time from injury to RTS increases by a factor of 1.02 days (exp{β}=1.02 days; 95% CI: 1.01, 1.02 days; p<0.001). For every one-unit increase in DHI score, the geometric mean of time from injury to RTS increases by a factor of 1.02 days (exp{β}=1.02 days; 95% CI: 1.01, 1.02 days; p<0.001). The R-squared for this model was 0.51, meaning that the model explains 51% of the total variance of time from injury to RTS.

Nomogram

**Figure 3:**
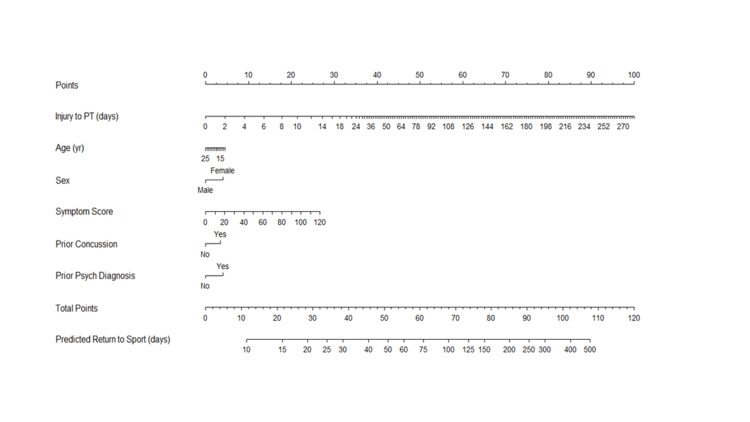
Nomogram for the predicted time from injury to return-to-sport (RTS). Nomogram for predicting the return-to-sport time following a concussion.

**Figure 4:**
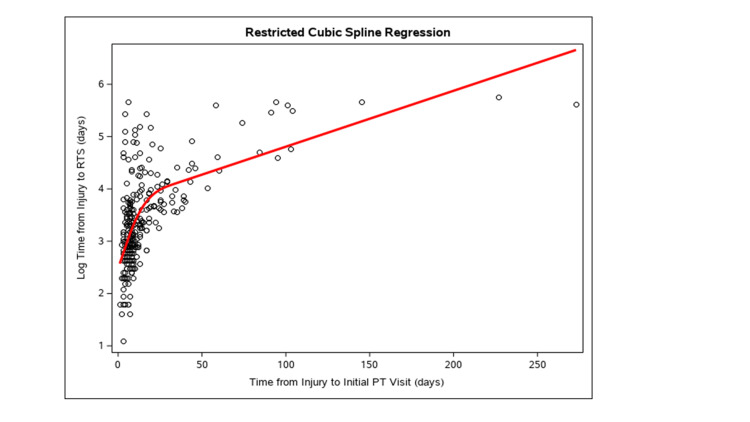
Restricted cubic spline regression of time from injury to initial PT visit to predict time from injury to return-to-sport (RTS). PT: Physical therapy A restricted cubic spline model was created to demonstrate the relationship between the time from injury and the initial physical therapy appointment to return to sport.

A nomogram (Figure 3) was created based on restricted cubic spline modeling (Figure 4) to demonstrate the relationship between predicted variables of interest and time to return-to-play. A nomogram allows for the estimation of the impact of various factors on the noted outcome.

## Discussion

The purpose of this study was to examine the relationship between early initiation of vestibular therapy following SRC and total time to RTP. The primary finding was that a positive association exists between time to initial vestibular therapy appointment and total time to RTP. The multivariate regression analysis demonstrated that for every one-day increase in time from injury to initial PT, the days to RTP increase by 1.02 days (Table 2). These findings were demonstrated in a population of adolescent and young adult athletes with a balance of a variety of sports represented in the population. Additionally, using restricted cubic spline modeling, it was demonstrated that a greater time until initial physical therapy intervention may be associated with exponentially increased time until return-to-play (Table 2). For example, an athlete presenting for an initial physical therapy evaluation at 20 days post-concussion could have a RTP time that increased by a factor of 1.6 days compared to if he or she had presented for PT at 10 days post-injury. Cubic spline modeling suggests that the impact of timing to PT initiation on RTP increases most significantly in the period immediately post-injury, with the degree of benefit beginning to decrease once outside of the first two to four weeks post-injury. Other research has shown that SRC with vestibular dysfunction is associated with prolonged recovery, but research has not identified whether early initiation of vestibular therapy can reduce the total time to recovery and RTP [2,18]. Using the above modeling, a nomogram was created to allow for the potential prediction of time from injury to RTS based on a variety of factors, including time from injury to initial PT.

Limitations

Additional research is needed to establish the association between early vestibular therapy further and return to sports after a sports-related concussion. More research is needed regarding the most beneficial time for initiation of vestibular therapy, duration of therapy, and frequency of therapy. Additionally, the patients in this study were all between 12 and 25 years of age and had suffered a sports-related concussion. The applicability of these findings to patients older or younger than this age range or those who suffered a concussion by a non-sports-related mechanism cannot be determined.

Clinical implications

The focus of concussion management has shifted towards specific therapies targeted at the various concussion pathology domains. Additionally, there has been a shift towards an early resumption of physical and cognitive activity after the injury as opposed to prolonged activity restriction [3]. However, to date, there had been limited evidence on the impact of early initiation of these targeted therapies. Our study demonstrates that early vestibular therapy may facilitate earlier RTP for adolescents and young adults with SRC. While the benefit of early initiation of vestibular therapy was relatively small, it should be noted that early initiation of targeted vestibular therapy was not deleterious to the athlete. Additionally, the RTP benefit of early initiation of vestibular therapy was most pronounced when therapy was initiated early (i.e. in the first days to weeks) in the post-injury course [4]. This stands in contrast to the previous model for managing vestibular symptoms, which often waited until symptoms had persisted for multiple weeks after the initial injury before consideration was given to initiation of directed therapy. At a minimum, a provider who recognizes symptoms of vestibular dysfunction in an athlete with a concussion can feel confident that targeted therapy is unlikely to be harmful and has the highest potential for RTP benefit when started early in the post-injury course. Directed therapy exercises also represent an opportunity to keep an athlete engaged in the concussion recovery process, giving them a greater sense of control over their injury and situation as opposed to more passively waiting for symptoms to abate [6]. The impact of early vestibular therapy on academic resumption and recovery was not formally addressed, but RTP typically follows recovery and tolerance for academic work. Overall, even a small reduction in time away from academics and athletics due to a concussion may be significant for many young athletes.

Additionally, through the data obtained in this study, a nomogram was created that allows for the projection of the recovery timeframe for an athlete with vestibular dysfunction after an SRC. This tool has two potential benefits. First, it allows for a visual representation of the general impact of a specific factor on return-to-play time and even relative impact within a specific range for variables represented on a continuous scale. For example, in Figure 4, the injury to PT factor demonstrates an increased impact of time to PT in the first 14 days given the increased point weight for the first 14 to 18 days approximately. Conversely, delays in PT initiation had progressively less impact as time continued. Additionally, the nomogram can be applied in specific athlete situations. For example, a 15-year-old female with no history of concussion or underlying psychiatric diagnosis presents with an initial symptom score of 40 and initiates physical therapy approximately 14 days post-injury. Her total score on the nomogram scale would be 46 points (calculated as 5 points for age, 5 for gender, 27 for time to PT, and 9 for symptom score). Using this total point score, her projected recovery timeframe would be approximately 40 days. Though there will understandably be some variation between projected and actual recovery for each individual, this tool may help to guide patient, parent, and coaching expectations for recovery timeframe after injury.

## Conclusions

The results of this research suggest that early initiation of vestibular therapy following SRC is associated with a reduction in recovery time and RTP. Recognition of vestibular dysfunction early in the course of an SRC should prompt clinicians to consider early initiation of vestibular therapy in order to maximize return-to-play benefits. The incorporation of vestibular therapy into a multidisciplinary concussion treatment protocol may reduce the overall time to recover and return to play. Future research and prospective trials to determine the optimal timing and frequency of therapy as well as generalizability to a broader population are needed. Additionally, the created nomogram is an additional tool that can be used for the projection of recovery timeframe based on the included factors, and additional research is needed to validate further and expand the applicability of the nomogram.
